# Transcriptomic Profiling of Two Rice Thermo-Sensitive Genic Male Sterile Lines with Contrasting Seed Storability after Artificial Accelerated Aging Treatment

**DOI:** 10.3390/plants13070945

**Published:** 2024-03-25

**Authors:** Fan Li, Hongbing Ye, Yingfeng Wang, Jieqiang Zhou, Guilian Zhang, Xiong Liu, Xuedan Lu, Feng Wang, Qiuhong Chen, Guihua Chen, Yunhua Xiao, Wenbang Tang, Huabing Deng

**Affiliations:** 1 College of Agronomy, Hunan Agricultural University, Changsha 410128, China; lifancd@163.com (F.L.); xy_abs@163.com (H.Y.); wangyingfeng229@163.com (Y.W.); crackzjq@163.com (J.Z.); zgl604@163.com (G.Z.); xiongliu@whu.edu.cn (X.L.); luxuedan1@126.com (X.L.); wangfenghifi@126.com (F.W.); cqh924@163.com (Q.C.); chenguihua1977@163.com (G.C.); yhxiao@hunau.edu.cn (Y.X.); 2 Hunan Provincial Key Laboratory of Rice and Rapeseed Breeding for Disease Resistance, Changsha 410128, China; 3 State Key Laboratory of Hybrid Rice, Hunan Hybrid Rice Research Center, Changsha 410128, China

**Keywords:** *Oryza sativa* L., TGMS line, seed storability, artificially accelerated aging treatment, transcriptome analysis

## Abstract

Seed storability has a significant impact on seed vitality and is a crucial genetic factor in maintaining seed value during storage. In this study, RNA sequencing was used to analyze the seed transcriptomes of two rice thermo-sensitive genic male sterile (TGMS) lines, S1146S (storage-tolerant) and SD26S (storage-susceptible), with 0 and 7 days of artificial accelerated aging treatment. In total, 2658 and 1523 differentially expressed genes (DEGs) were identified in S1146S and SD26S, respectively. Among these DEGs, 729 (G1) exhibited similar regulation patterns in both lines, while 1924 DEGs (G2) were specific to S1146S, 789 DEGs (G3) were specific to SD26S, and 5 DEGs (G4) were specific to contrary differential expression levels. Gene Ontology (GO) and Kyoto Encyclopedia of Genes and Genomes (KEGG) analysis showed that “translation”, “ribosome”, “oxidative phosphorylation”, “ATP-dependent activity”, “intracellular protein transport”, and “regulation of DNA-templated transcription” were significantly enriched during seed aging. Several genes, like *Os01g0971400*, *Os01g0937200*, *Os03g0276500*, *Os05g0328632*, and *Os07g0214300*, associated with seed storability were identified in G4. Core genes *Os03g0100100* (*OsPMEI12*), *Os03g0320900* (*V2*), *Os02g0494000*, *Os02g0152800*, and *Os03g0710500* (*OsBiP2*) were identified in protein–protein interaction (PPI) networks. Seed vitality genes, *MKKK62* (*Os01g0699600*), *OsFbx352* (*Os10g0127900*), *FSE6* (*Os05g0540000*), and *RAmy3E* (*Os08g0473600*), related to seed storability were identified. Overall, these results provide novel perspectives for studying the molecular response and related genes of different-storability rice TGMS lines under artificial aging conditions. They also provide new ideas for studying the storability of hybrid rice.

## 1. Introduction

Rice (*Oryza sativa* L.) is a staple food crop in China and worldwide. Hybrid rice, a significant innovation, has made a substantial contribution to the stable and high yield of rice [1,2]. A thermo-sensitive genic male sterile (TGMS) line is the basis for successful two-line hybrid rice breeding; it can be not only used as a female parent to cross with the restorer line to produce hybrid seeds, but also propagated by self-crossing [2,3,4]. The seed storability of hybrid rice seeds is different from that of conventional rice seeds. In southern China, the germination rate of hybrid rice seeds can drop below 70% after one year of storage, resulting in significant economic losses [5]. To slow down the process of seed aging and deterioration of rice grain quality, excess hybrid seeds need to be stored under appropriate conditions. It is important to note that seed storability varies among hybrid rice varieties. In fact, the F_1_ hybrid seeds are the same as sterile line seeds. The storability of hybrid seeds depends mainly on the varieties of sterile lines, their developmental status, and storage conditions [6,7]. Therefore, it is essential to investigate and enhance the seed storability of TGMS lines to ensure the safe and high-quality production of hybrid rice.

Seed storability refers to the capacity of mature seeds to maintain their original seed vigor after long-term storage. Physiological changes inevitably occur inside the seed during storage, resulting in a decline or loss of seed vitality [8]. In southern China, rice seeds are usually stored in energy-consuming ways, such as using storage warehouses with controllable temperature and humidity or building low-temperature closed grain warehouses filled with inert gases, which also cause many environmental problems [9]. Therefore, enhancing the seed storability of TGMS lines is an economical and environmentally friendly way to cope with the surplus of hybrid seeds.

Seeds undergo a series of physiological and biochemical reactions during aging, including the degradation of soluble sugars and proteins, disturbances in gene expression, and nucleic acid degradation. Ribosomal RNA integrity is closely correlated with seed aging, and it can serve as a rapid indicator of seed aging [10]. Ribosomes are indispensable components of cells that translate mRNA into functional proteins [11]. Endogenous signals and environmental stimuli, such as ambient temperature, can regulate ribosome biosynthesis [12,13,14]. Previous studies have identified that low temperature rapidly inhibits ribosomal biogenesis [15]. High-temperature conditions during seed aging also have a significant impact on ribosome synthesis and processing. Transcription and translation are two key steps in the process of gene expression. Transcriptional regulation plays a crucial role in seed vigor, dormancy, and germination [16,17]. The translation process is inextricably linked to the ribosome, where it primarily occurs, and the loss of translational function directly leads to a decline in seed vigor [18]. Additionally, seed aging up-regulates the expression of genes associated with the oxidative phosphorylation pathway, which leads to the generation of reactive oxygen species (ROS), further aggravating seed aging [19,20].

Seed longevity is directly determined by the level of internal energy storage and metabolism [21]. Reduced seed viability can be caused by redox imbalance and reduced efficiency of seed energy production due to seed aging [22]. Mitochondria play a crucial role in seed aging. The aging process damages the membrane, leading to a loss of mitochondrial function and a reduction in ATP supply [23,24]. All ATP-dependent activities are inhibited during seed aging. Seed aging also results in a burst of ROS that induces alterations and modifications in certain mitochondrial proteins and intracellular transport, ultimately resulting in a loss of seed viability [25]. Changes in protein indicate alterations in genetic stability, a decrease in storage protein content, and an increase in soluble protein content in seeds during aging. Protein processing activities were also enriched during seed storage but down-regulated due to seed aging [26,27].

Seed aging is associated with other factors, such as plant phytohormones [9]. Abscisic acid (ABA) and gibberellin (GA) are recognized as key phytohormones in regulating seed dormancy and germination [21]. During seed aging, genes related to plant hormone signal transduction are up-regulated, and ABA signaling is activated, resulting in the maintenance of seed viability and longevity [28]. The crosstalk between ABA and ethylene (ET) [29,30], as well as ABA and cytokinin (CK) [31,32], plays a crucial role in a plant’s response to abiotic stress. Additionally, seed aging is often accompanied by a decrease in enzymatic activity [8,33]. Increased ROS lead to the inactivation of vital enzymes in ATP synthesis and Calvin cycle processes, causing cellular dysfunction [34]. Enzymatic systems in plants are essential for scavenging intracellular ROS and delaying seed aging. Previous studies have investigated the factors that influence seed storage tolerance. However, there are still several unexplained issues.

Seed storability is a complex trait that is strictly regulated by multiple factors and involves various mechanisms and pathways [35]. Further exploration is required to identify additional regulatory mechanisms and genes related to seed storability. Although natural aging poses challenges for studying seed storability, artificial accelerated aging can be used to accelerate the aging process under controlled conditions and cause seeds to lose vigor rapidly, thereby significantly reducing processing time [36,37]. In this study, we conducted an investigation into transcriptomes of S1146S and SD26S at 0 and 7 days of seed aging treatment using high-throughput RNA sequencing (RNA-seq) technology. The aim was to analyze the complicated molecular responses occurring at the transcriptional level in these TGMS lines under seed aging stress conditions. The results of this study will contribute to the interpretation of complicated regulatory mechanisms and identify key genes associated with the seed aging stress response of TGMS lines.

## 2. Results

### 2.1. TGMS Line S1146S Had Better Seed Storability Than SD26S

**Figure 1:**
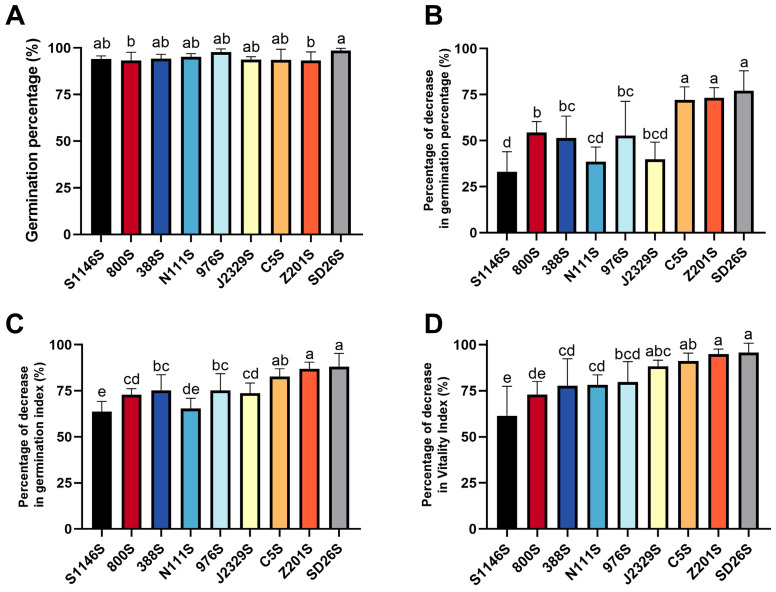
Seed storability of 9 thermo-sensitive genic male sterile (TGMS) lines. (**A**) The final germination percentage of 9 TGMS lines on the 14th day in standard germination experiments. (**B**) The decrease in gemination percentage (GP), (**C**) germination index (GI), and (**D**) vitality index (VI) of 9 TGMS lines at 14 d artificial accelerated aging treatment. Different letters indicate statistical differences between samples using ANOVA test, *p* < 0.05.

To characterize the difference in seed storability among TGMS lines S1146S, SD26S, 800S, 388S, N111S, 976S, J2329S, C5S, and Z201S, we calculated the germination percentage (GP), germination index (GI), and vitality index (VI) of nine TGMS lines before and after artificial accelerated aging treatment. The initial GP was highest at 98.5% for SD26S and 94% for S1146S, with no significant difference between them (Figure 1A). After artificial accelerated aging treatment for 14 days, the GP, GI, and VI of the nine TGMS lines decreased to varying degrees. S1146S had the least decrease in GP at 33.1%, followed by N111S and J2329S which showed decreases of 38.6% and 39.9% in GP, respectively. However, SD26S showed the greatest decrease of up to 77.0% (Figure 1B). The GI of S1146S and N111S decreased by 63.6% and 65.4%, respectively, with the largest decrease of 88.1% in SD26S (Figure 1C). S1146S showed the least decrease in VI at 61.4%, followed by 800S and 388S at 73.0% and 77.8%, respectively, and SD26S had the highest decrease in VI at 95.7% (Figure 1D). In conclusion, the percentage decreases in GP, GI, and VI of S1146S were much lower than those of SD26S after artificial aging treatment. Compared with SD26S, artificially accelerated aging was less detrimental to the seed viability of S1146S, indicating that S1146S has better seed storability than SD26S.

### 2.2. DEG Analysis and qRT-PCR Verification

Seeds aged for 14 days underwent artificial accelerated aging, which severely impaired their viability and made it difficult to obtain RNA. Therefore, we used seeds aged for 7 days as RNA-seq material. S1146S and SD26S seeds were subjected to 45 °C and 90–95% relative humidity (RH) for 7 days, while untreated seeds were used as controls. As a result, S1146S and SD26S each had one treatment (7 d) and one control (0 d). RNA-seq was performed on 12 samples, each with over 6 G of sequencing data and a high mapping rate (Table S1).

**Figure 2:**
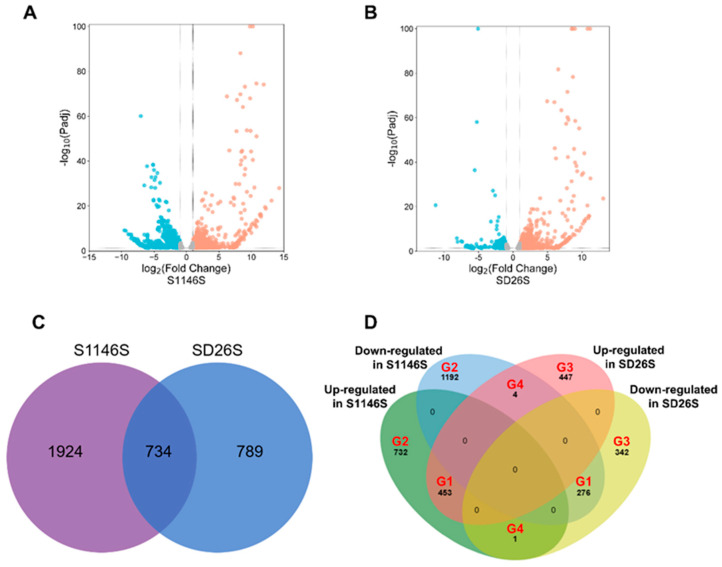
Statistical analysis of differentially expressed genes (DEGs) in S1146S and SD26S. The relationship between fold change and false discovery rate (FDR) of DEGs in (**A**) S1146S and (**B**) SD26S is shown by volcano plots. The blue dots indicate down-regulated DEGs, the orange dots indicate up-regulated DEGs, and the grey dots indicate not sig. (**C**) Venn diagram comparison of total DEGs in S1146S and SD26S. (**D**) Venn diagram demonstrating the overlap of DEGs in S1146S and SD26S. Specifically, G1 represents the DEGs with similar expression in both S1146S and SD26S, G2 represents the DEGs specific to S1146S, G3 represents the DEGs specific to SD26S, and G4 represents DEGs that were oppositely expressed in S1146S and SD26S.

After aging treatment, 2658 differentially expressed genes (DEGs) were identified in S1146S, with 1186 up- and 1472 down-regulated genes (Figure 2A). Meanwhile, 1523 DEGs were identified in SD26S, with 904 up- and 619 down-regulated genes (Figure 2B). The number of DEGs in the storage-tolerant variety S1146S was higher than that in the storage-susceptible variety SD26S. S1146S had more down-regulated genes than up-regulated genes, while the opposite was true for SD26S. S1146S and SD26S co-expressed 734 DEGs; 1924 DEGs were unique to S1146S, and 789 DEGs were unique to SD26S (Figure 2C).

To analyze the difference in DEGs between S1146S and SD26S, the DEGs were classified. A total of 3447 unique DEGs were divided into four groups (G1, G2, G3, and G4) (Figure 2D). In G1, DEGs were similarly expressed in both the storage-tolerant variety S1146S and the storage-susceptible variety SD26S, with 729 DEGs identified (453 up- and 276 down-regulated) (Table S2). DEGs were specific to the storage-tolerant variety S1146S in G2, with 1924 (732 up- and 1192 down-regulated) (Table S2). In G3, 789 DEGs (447 up- and 342 down-regulated) were specific to the storage-susceptible variety SD26S (Table S2). Notably, DEGs in the storage-tolerant variety S1146S and the storage-susceptible variety SD26S were subject to opposite regulation in G4. It contained five DEGs, with one being up-regulated in S1146S and down-regulated in SD26S, while the other four genes were down-regulated in S1146S and up-regulated in SD26S (Table S2).

To verify the reliability of the sequencing results, we randomly selected six representative genes and verified the changes in expression levels through quantitative real-time PCR (qRT-PCR) for 0 and 7 days of seed aging treatment. The validation results are shown in Figure S1, which shows that the relative trends of the expression profiles in the qRT-PCR were consistent with the RNA-seq data.

### 2.3. Gene Ontology and Pathway Enrichment Analysis of DEGs

**Figure 3:**
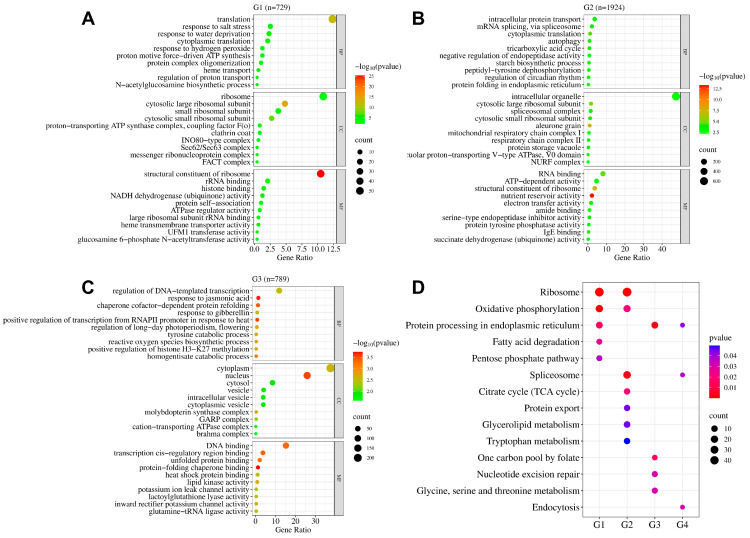
Functional annotation of DEGs in G1, G2, G3, and G4. (**A**–**C**) Gene Ontology (GO) annotation of DEGs in G1, G2, and G3. The horizontal axis represents the gene ratio of DEGs annotated to each specific function, and the vertical axis represents the functional annotation information. (**D**) Comparison of Kyoto Encyclopedia of Genes and Genomes (KEGG) pathway enrichment in G1, G2, G3, and G4. The dot color represents the *p*-value; the smaller the *p*-value, the closer the color is to red. The dot size reflects the relative number of DEGs related to each pathway.

The enriched DEGs in groups G1, G2, G3, and G4 were classified by GO annotation. Our analysis identified 651 GO terms, including various biological processes (BPs), cellular components (CCs), and molecular functions (MFs). In particular, 350 GO terms were related to BPs, 101 to CCs, and 200 to MFs. The top 10 GO terms for each of the three categories in each group are presented in Figure 3A–C.

The expression of DEGs in the G1 group was similar in both the storage-tolerant variety S1146S and the storage-susceptible variety SD26S. The prominent GO terms included “translation” (GO:0006412, BP), “ribosome” (GO:0005840, CC), “structural constituent of ribosome” (GO:0003735, MF), “cytosolic large ribosomal subunit” (GO:0022625, CC), and “small ribosomal subunit” (GO:0015935, CC) (Figure 3A, Table S3).

The G2 group comprised DEGs specific to the storage-tolerant variety S1146S. The GO terms in this group encompassed “intracellular organelle” (GO:0043229, CC), “RNA binding” (GO:0003723, MF), “ATP-dependent activity” (GO:0140657, MF), “intracellular protein transport” (GO:0006886, BP), and “structural constituent of ribosome” (GO:0003735, MF) (Figure 3B, Table S4).

The G3 group comprised DEGs specific to the storage-susceptible variety SD26S. The most prominent GO terms included “cytoplasm” (GO:0005737, CC), “nucleus” (GO:0005634, CC), “DNA binding” (GO:0003677, MF), “regulation of DNA-templated transcription” (GO:0006355, BP), and “cytosol” (GO:0005829, CC) (Figure 3C, Table S5).

The DEGs of the G4 group exhibited opposite regulation in the storage-tolerant variety S1146S and the storage-susceptible variety SD26S. Notably, GO terms in this group encompassed “proteolysis” (GO:0006508, BP), “protein catabolic process” (GO:0030163, BP), “endopeptidase activity” (GO:0004175, MF), “extracellular space” (GO:0005615, CC), and “vacuole” (GO:0005773, CC) (Figure S2A, Table S6). Further analysis revealed that only one DEG (*Os01g0971400*) was up-regulated in S1146S and down-regulated in SD26S, and it was related to “proteolysis” (GO:0006508, BP) (Figure S2B, Table S6). Meanwhile, four DEGs (*Os01g0937200*, *Os03g0276500*, *Os05g0328632*, *Os07g0214300*) were generally down-regulated in S1146S and up-regulated in SD26S (Figure S2C–F). *Os01g0937200* and *Os07g0214300* were also related to “proteolysis” (GO:0006508, BP). *Os03g0276500* was involved in “cellular response to unfolded protein” (GO:0034620, BP). *Os05g0328632* was related to “vacuole” (GO:0005773, CC) (Table S6). The difference in the expression changes of *Os01g0971400*, *Os01g0937200*, *Os03g0276500*, *Os05g0328632*, and *Os07g0214300* between S1146S and SD26S might be a contributing factor to the difference in seed storability.

To further determine whether seed storability DEGs were involved in specific pathways, KEGG pathway analysis was performed for the DEGs in groups G1, G2, G3, and G4. The analysis showed that 245 DEGs were involved in 14 pathways (Table S7).

The G1 group comprised DEGs that were similarly expressed in both the storage-tolerant variety S1146S and the storage-susceptible variety SD26S. The top KEGG terms were “ribosome” (ko03010), “oxidative phosphorylation” (ko00190), “protein processing in endoplasmic reticulum” (ko04141), “fatty acid degradation” (ko00071), and “pentose phosphate pathway” (ko00030) (Figure 3D, Table S7).

In G2, DEGs specific to the storage-tolerant variety S1146S were identified. The KEGG terms “ribosome” (ko03010), “spliceosome” (ko03040), “citrate cycle (TCA cycle)” (ko00020), “oxidative phosphorylation” (ko00190), “protein export” (ko03060), “glycerolipid metabolism” (ko00561), and “tryptophan metabolism” (ko00380) were prominent KEGG terms in this group (Figure 3D, Table S7).

The G3 group consisted of DEGs specific to the storage-susceptible variety SD26S. Notable KEGG terms in this group included “protein processing in endoplasmic reticulum” (ko04141), “one carbon pool by folate” (ko00670), “nucleotide excision repair” (ko03420), and “glycine, serine and threonine metabolism” (ko00260) (Figure 3D, Table S7).

The G4 group included DEGs with opposite expression profiles in the storage-tolerant variety S1146S and the storage-susceptible variety SD26S. The prominent KEGG terms in this group included “endocytosis” (ko04144), “spliceosome” (ko03040), and “protein processing in endoplasmic reticulum” (ko04141) (Figure 3D, Table S7).

Interestingly, we observed that some pathways were common to different groups, such as “ribosome” (ko03010) and “oxidative phosphorylation” (ko00190) between the G1 and G2 groups, “spliceosome” (ko03040) between the G2 and G4 groups, and “protein processing in the endoplasmic reticulum” (ko04141) between the G1, G3, and G4 groups (Figure 3D, Table S7).

### 2.4. Key Pathways Related to Seed Aging Treatment

The GO and KEGG enrichment analysis showed that DEGs related to “translation”, “ribosome”, and “oxidative phosphorylation” were significantly enriched in the G1 group (Figure 3A,D). DEGs particularly enriched in the G2 group were associated with “ATP-dependent activity” and “intracellular protein transport” (Figure 3B). Meanwhile, DEGs specifically enriched in the G3 group were associated with “regulation of DNA-templated transcription” (Figure 3C). To investigate the correlation between the differences in the regulation of these pathways during seed aging and the seed storability of the two varieties, we drew a heatmap of the expression of DEGs associated with each pathway.

As can be seen in the heatmap of the related genes involved in “ribosome”, “translation”, and “oxidative phosphorylation”, DEGs had the same regulation trends between S1146S and SD26S, and most DEGs were induced to be up-regulated after seed aging, with S1146S showing a higher degree of up-regulation than SD26S (Figure S3A–C). The results indicated an activation tendency in ribosome-related, translation-related, and oxidative phosphorylation-related activities during seed aging of S1146S and SD26S.

**Figure 4:**
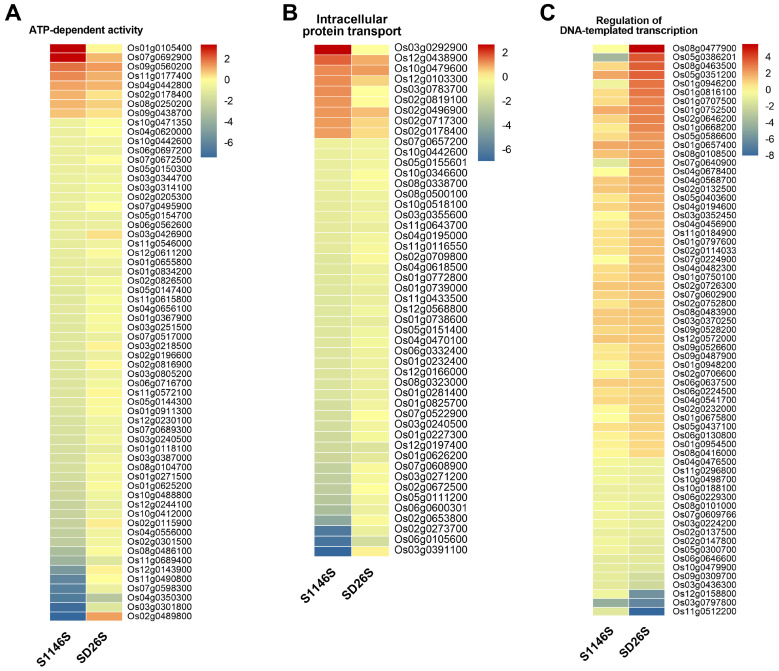
Key pathways related to seed aging in S1146S and SD26S. Heatmap depicting the expression levels of the DEGs enriched in the (**A**) “ATP-dependent activity” term, (**B**) “Intracellular protein transport” term, and (**C**) “regulation of DNA-templated transcription” term. The log_2_ fold change (log_2_ FC) values of all samples were used to construct the heatmap. The gene expression levels are represented by a blue to red color spectrum, indicating low to high expression, respectively.

In the heatmap related to “ATP-dependent activity”, 8 DEGs were induced to be up-regulated, namely *Os01g0105400*, *Os07g0692900*, *Os09g0560200*, *Os11g0177400*, *Os04g0442800*, *Os02g0178400*, *Os08g0250200*, and *Os09g0438700*, while the remaining 53 DEGs were suppressed after seed aging treatment in S1146S. However, the regulation trends of DEGs in SD26S were not obvious (Figure 4A). The DEGs associated with “intracellular protein transport” exhibited similar regulation patterns. Among these, 9 DEGs, namely *Os03g0292900*, *Os12g0438900*, *Os10g0479600*, *Os12g0103300*, *Os03g0783700*, *Os02g0819100*, *Os02g0496900*, *Os02g0717300*, and *Os02g0178400*, were induced to be up-regulated, while the remaining 40 DEGs were suppressed after seed aging treatment in S1146S (Figure 4B). These findings indicated that these specific DEGs were up-regulated in response to “ATP-dependent activity” and “intracellular protein transport” and actively participated in the response to seed aging treatment in the storage-tolerant genotype S1146S.

Among the DEGs associated with the “regulation of DNA-templated transcription”, 48 DEGs were induced to be up-regulated in SD26S, including *Os08g0477900*, *Os05g0386201*, *Os08g0463500*, *Os05g0351200*, *Os01g0946200*, *Os01g0816100*, *Os01g0707500*, and *Os01g0752500*. No clear trend was observed in S1146S (Figure 4C). The results suggested that these specific DEGs, which were induced to be up-regulated and associated with “regulation of DNA-templated transcription”, actively participated in the response of the storage-susceptible genotype SD26S to seed aging treatment.

### 2.5. PPI Network Construction and Core Gene Analysis of DEGs

**Figure 5:**
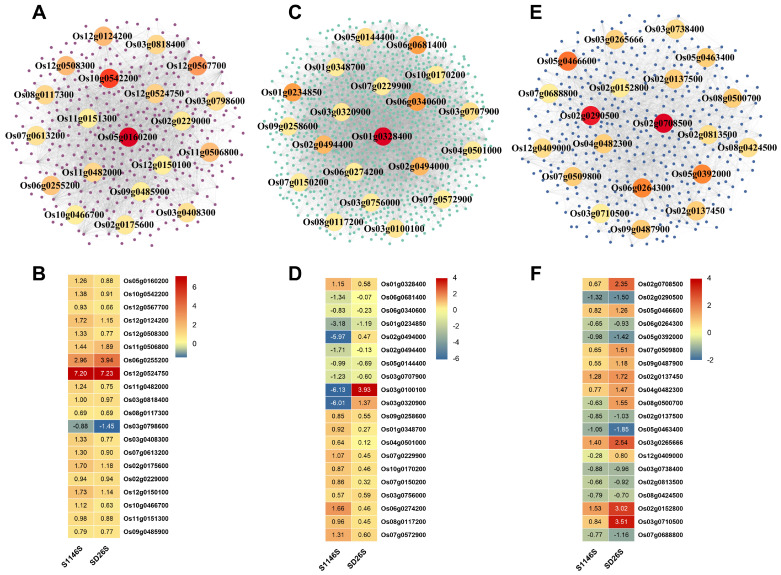
Protein–protein interaction (PPI) analysis of G1, G2, and G3 DEGs. PPI networks for G1 (**A**), G2 (**C**), and G3 (**E**) DEGs were constructed from the STRING database. Genes are shown as dots, and their relationships are shown as lines. The top 20 DEGs in the interaction network were calculated based on their degree, with darker colors indicating greater degrees. The log_2_ FC of the top 20 DEGs in the G1 (**B**), G2 (**D**), and G3 (**F**) PPI networks are represented in the heatmaps. The number indicates the log_2_ FC value of the DEGs at the corresponding time point.

Protein–protein interaction (PPI) networks were constructed for G1, G2, and G3 DEGs to identify core genes. The top 20 DEGs with the highest degree of protein interaction were shown in the PPI networks of G1, G2, and G3 (Figure 5A,C,E). The heatmap depicted the expression patterns of these genes. In G1, all genes were induced to be up-regulated except for *Os03g0798600*, and these genes were mainly ribosomal protein genes associated with translation (Figure 5B). In G2, three genes were significantly down-regulated in S1146S, namely *Os03g0100100* (*OsPMEI12*)*, Os03g0320900* (*V2*), and *Os02g0494000*, which were associated with pectin esterase inhibitor activity, guanylate kinase activity, and binding, respectively (Figure 5D). From the heatmap of G3, we concluded that unfolded protein response-related genes *Os02g0152800* and *Os03g0710500* (*OsBiP2*) were significantly up-regulated in SD26S (Figure 5F).

### 2.6. Comparative Analysis of the Expression Levels of “Seed Vitality-Related Genes” in S1146S and SD26S

**Figure 6:**
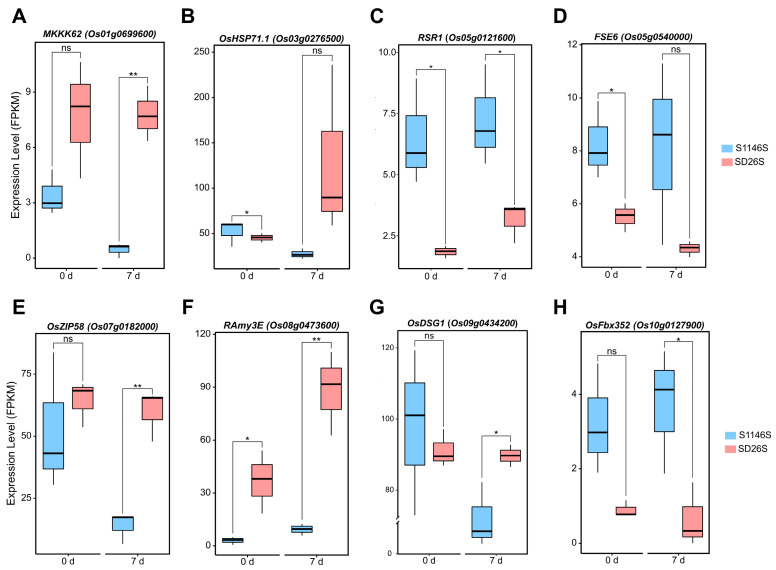
Comparative analysis of the expression levels of genes associated with “seed vitality” between S1146S and SD26S after artificial seed aging treatment for 0 and 7 days. Comparative analysis of (**A**) *MKKK62* (*Os01g0699600*), (**B**) *OsHSP71.1* (*Os03g0276500*), (**C**) *RSR1* (*Os05g0121600*), (**D**) *FSE6* (*Os05g0540000*), (**E**) *OsZIP58* (*Os07g0182000*), (**F**) *RAmy3E* (*Os08g0473600*), (**G**) *OsDSG1* (*Os09g0434200*), (**H**) *OsFbx352* (*Os10g0127900*) expression levels (FPKM: fragments per kilobase of transcript per million mapped reads). The means ± SEM (standard error of the mean) of three independent replicates are presented as data. Statistical significance is denoted as follows: ns (not significant) for *p* ≥ 0.05, * for *p* < 0.05, and ** for *p* < 0.01.

To further investigate the reasons for the differences in seed storability between S1146S and SD26S, we compared the expression levels of “seed vitality-related genes” in the storage-tolerant genotype S1146S and the storage-susceptible genotype SD26S. Among these DEGs, we identified *MKKK62* (*Os01g0699600*), which encodes a mitogen-activated protein kinase that negatively regulates rice seed dormancy, and overexpression of *MKKK62* results in the disruption of seed dormancy and regulates seed dormancy by altering *OsMFT* transcription [38]. Our results revealed that the expression level of *MKKK62* in SD26S was higher than that of in S1146S at 0 and 7 days of seed aging treatment (Figure 6A). *OsFbx352* (*Os10g0127900*) regulates glucose-induced inhibition of seed germination by affecting ABA synthesis and catabolism [39]. In our study, expression levels of the *OsFbx352* gene were higher in S1146S under normal storage conditions and seed aging (Figure 6H). Additionally, the expression of the *Floury Shrunken Endosperm6* (*FSE6*) gene in SD26S was lower than that of S1146S after 0 and 7 days of aging treatment (Figure 6D). *FSE6* encodes a glycosyltransferase that is homologous to *Arabidopsis* GINT1 and is related to cellulose content and starch biosynthesis [40]. The *RAmy3E* gene encodes a α-amylase isoform 3E, a key enzyme in the breakdown of starch during germination of cereal seeds [41,42]. Our findings revealed that the expression level of *RAmy3E* in SD26S was significantly higher than that in S1146S, and seed aging treatment increased this difference between them (Figure 6F).

In addition, several genes, including *OsHSP71.1* (*Os03g0276500*), *RSR1* (*Os05g0121600*), *OsZIP58* (*Os07g0182000*), and *OsDSG1* (*Os09g0434200*), exhibited exclusive differential expression levels between S1146S and SD26S (Figure 6B,C,E,G). *OsHSP71.1* encodes rice heat shock proteins, and its expression is induced by ABA, which may be involved in the acquisition of rice seed desiccation tolerance [43,44]. *RSR1* and *OsZIP58* impact seed vitality through their involvement in the regulation of starch biosynthesis [43]. *OsDSG1* encodes a RING finger E3 ubiquitin ligase that binds to OsABI3 and regulates the ABA signaling pathway during seed dormancy and germination [45]. These findings suggest that the DEGs related to “seed vitality” have a significant impact on seed storability.

## 3. Discussion

Hybrid seed storability is influenced by genetic and environmental factors, mainly by genetic factors, and determined by the varieties of rice sterile lines [6,46]. Breeding rice sterile lines with strong storability is the most cost-effective way to solve the problem of hybrid rice seed storage. In this study, we characterized the germination percentage, germination index, and vitality index of nine TGMS lines before and after seed aging treatment (Figure 1). In comparison to the sensitive variety SD26S, it was observed that S1146S had better seed storability after seed aging treatment. The number of DEGs was higher in S1146S than in SD26S (Figure 2C), which might suggest that the storage-tolerant line S1146S underwent a more complex response than the storage-susceptible SD26S during aging stress. This finding differs from previous studies [47]. Identifying the key mechanisms of seed aging response in the storage-tolerant line S1146S will help us to introduce its storability into the breeding of superior hybrid rice.

Proteins and sugars are the two main storage substances of seeds. Changes in proteins can reflect the variations in genetic stability. Soluble proteins and soluble sugars play key roles in regulating seed storage properties and viability [35,48]. The seed storability is also influenced by the balance between ROS production and the antioxidant system [49]. Mitochondria serve as the energy supply center of the cell, and aging disrupts mitochondrial function, leading to an inadequate supply of ATP and accumulation of ROS [50,51]. Our findings suggest that common and genotype-specific mechanisms related to seed storability existed in both tolerant and susceptible varieties. To further analyze the difference in DEGs between S1146S and SD26S, we classified 3447 DEGs into four groups (Figure 2D) and performed GO and KEGG enrichment analysis. This study found that DEGs related to “translation”, “ribosome”, and “oxidative phosphorylation” were significantly enriched in the G1 group (Figure 3A,D). DEGs associated with “ATP-dependent activity” and “intracellular protein transport” were specifically enriched in the G2 group (Figure 3B), while DEGs particularly enriched in the G3 group were associated with “regulation of DNA-templated transcription” (Figure 3C).

Ribosomes are closely related to seed storage and germination. They are necessary for restarting translational and post-transcriptional processing associated with stress responses and developmental processes in stored seeds [52]. Additionally, the integrity of ribosomal RNA is closely related to seed aging and can be used as a rapid indicator of seed aging [10]. The translation process primarily occurs on ribosomes. Studies have demonstrated that seed aging treatment results in a reduction in translation capacity, leading to a decrease in seed vigor [18]. Oxidative phosphorylation pathways were stimulated and up-regulated in *Metasequoia glyptostroboides* seed during seed aging [19]. Repairing of misfolded proteins in the endoplasmic reticulum (ER) requires a significant amount of energy from the oxidative phosphorylation pathway, and this process can further aggravate seed aging as the mitochondria generate harmful ROS while providing ATP [19,20]. Our study revealed that the regulation patterns of DEGs in S1146S and SD26S were identical in terms of “translation”, “ribosomes”, and “oxidative phosphorylation”, and most of the DEGs were induced to be up-regulated after seed aging (Figure S3A–C). This suggested that seed aging may affect “translation”, “ribosome”, and “oxidative phosphorylation” and induce the expression of genes related to these pathways.

Eight and nine differentially expressed genes (DEGs) were up-regulated in G2-enriched DEGs specific to the storage-tolerant variety S1146S, associated with “ATP-dependent activity” and “intracellular protein transport”, respectively (Figure 4A,B). Seed aging can cause membrane damage, leading to a loss of mitochondrial function and impaired ATP supply, which can inhibit ATP-related activity in the cell [23]. Seed aging can lead to a burst of ROS, inducing alterations in mitochondrial metabolism and intracellular transport, ultimately resulting in the loss of seed viability [25]. Nevertheless, relevant DEGs were specifically induced to be activated in S1146S, suggesting that these genes might be involved in maintaining seed viability during seed aging (Figure 4A,B). “Regulation of DNA-templated transcription” is significantly enriched during seed dormancy release and germination [53,54]. Research has shown that the biological process of “DNA template transcriptional regulation” is important in seed aging [28]. Our results suggested that the majority of DEGs associated with “regulation of DNA-templated transcription” were significantly up-regulated in the sensitive variety SD26S, which may explain its storage sensitivity (Figure 4C).

Notably, the PPI networks constructed for G1, G2, and G3 DEGs contain several core genes. In G1, the genes were involved in the ribosome and protein translation process (Figure 5A,B). In G2, three genes were significantly down-regulated in S1146S, namely *Os03g0100100* (*OsPMEI12*), *Os03g0320900* (*V2*), and *Os02g0494000*. These genes are associated with pectin esterase inhibitor activity, guanylate kinase activity, and binding, respectively (Figure 5C,D). Several studies have revealed the important functions of pectin methylesterase inhibitors (PMEIs) in response to abiotic stresses. In rice, the PMEI gene shows specific transcriptional regulatory responses to drought stress, salt stress, cold, and anaerobic conditions [55]. Mutations in the PMEI gene (*At1g62760*) reduced sensitivity to salt stress, whereas overexpression of *AtPMEI5* increased seed germination and was detrimental to seed dormancy [56,57]. The down-regulation of *Os03g0100100* (*OsPMEI12*) in S1146S could be associated with high-temperature and high-humidity environmental stress during seed aging treatment. Additionally, *Os03g0320900* (*V2*) encodes a guanylate kinase localized to chloroplasts and mitochondria and is a key enzyme in the guanine metabolic pathway [58]. It has been shown that binding-related pathways are significantly enriched in maize during seed aging [31]. Furthermore, in times of stress, the endoplasmic reticulum experiences a rapid build-up of unfolded proteins, which triggers an unfolded protein response. This response leads to the release of Ca^2+^ into the cytoplasm, resulting in the accumulation of ROS in the mitochondria [19]. In G3, two genes related to the unfolded protein response, *Os02g0152800* and *Os03g0710500* (*OsBiP2*), were significantly up-regulated in SD26S (Figure 5F). These results suggest that seed aging may severely affect the normal function of the endoplasmic reticulum in SD26S seeds, resulting in the production of excessive amounts of unfolded proteins.

Five differentially expressed genes (DEGs) were identified in both lines from the G4 group, exhibiting opposite regulation patterns. Only one DEG (*Os01g0971400*) was found to be up-regulated in S1146S and down-regulated in SD26S, while the other four DEGs (*Os01g0937200, Os03g0276500, Os05g0328632,* and *Os07g0214300*) were down-regulated in S1146S and up-regulated in SD26S (Figure S2B–F). *Os01g0971400*, *Os01g0937200*, and *Os07g0214300* were related to “proteolysis”, *Os03g0276500* was related to “cellular response to unfolded protein”, and *Os05g0328632* was related to “vacuole” (Figure S2A, Table S6). Changes in proteins can reflect alterations in genetic stability during seed aging. Protein metabolism is closely related to the decline in seed viability caused by seed aging [59]. Similarly, unfolded proteins are products of the endoplasmic reticulum being overwhelmed by stress during seed aging [60]. The vacuole is an important site for protein storage in the seed endosperm. Large amounts of seed storage proteins are retained in the protein storage vacuole until germination [61]. Therefore, further exploration and research is required to determine the important roles that *Os01g0971400*, *Os01g0937200*, *Os03g0276500*, *Os05g0328632*, and *Os07g0214300* may play in the storability of S1146S and SD26S.

Several seed vitality-related genes were found in DEGs during seed aging treatment, including *MKKK62* (*Os01g0699600*) [38], *OsFbx352* (*Os10g0127900*) [39], *FSE6* (*Os05g0540000*) [40], and *RAmy3E* (*Os08g0473600*) [41,42]. The analysis of gene expression levels showed that *MKKK62* and *RAmy3E* were expressed at higher levels in SD26S compared to S1146S, which may be more conducive to reducing the seed dormancy of SD26S and thereby reducing its storability (Figure 6A,F). *FSE6* and *OsFbx352* were expressed at significantly higher levels in S1146S, which could contribute to enhanced seed viability of S1146S (Figure 6D,H). These findings may partly explain why S1146S exhibited stronger seed storability than SD26S.

In subsequent studies, it is necessary to create relevant transgenic materials to further analyze the exact role of these genes in seed storability. Meanwhile, S1146S can be used as an excellent germplasm resource for breeding seed-storage-tolerant rice TGMS lines, thereby improving the seed storability of two-line hybrid rice.

## 4. Materials and Methods

### 4.1. Plant Materials

TGMS lines S1146S, 800S, 388S, N111S, 976S, J2329S, C5S, Z201S, and SD26S were cultivated in the experimental field of Hunan Agricultural University in Baoshan, Yunnan in 2022. The materials were harvested at the same maturity level and immediately dried in an oven (101–34B, TAISETE, Tianjin, China) at 42 °C for 5 days to break dormancy and ensure a consistent germination percentage above 80%. The moisture content was then balanced to around 12%.

### 4.2. Artificial Aging Treatment

This study conducted artificial aging treatments to rapidly measure the storability of the seeds. The treatment method of Zeng et al. [62] was adopted with some modifications. After dormancy was broken, the seeds were incubated in a thermostatic moisture regulator (HCP153, Memmert, Germany) at 45 °C and 90–95% RH. Samples were harvested at 0 days, 7 days, and 14 days after the treatment.

### 4.3. Determination of Seed Storability

The standard germination test method was used. Seeds were soaked in water at 30 °C for 12 h after dormancy was broken, and the artificially aged seeds did not need to be soaked. For each treatment, 300 seeds were randomly counted and divided into three replicates, with 100 seeds per replicate. The seeds were placed in a germination box fitted with a special germination paper bed and incubated in a light- and temperature-controlled environment at 30 °C with 12 h of light and 12 h of darkness to promote germination. Care was taken to maintain consistent moisture levels in each germination box.

The daily count of germination (number of normal seedlings) was recorded. The day of placement into the incubator was considered as day 0. The germination test was concluded on the 14th day or when the maximum germination percentage was reached. The above-ground parts of the seedlings were then cut off, dried, and weighed. The germination percentage (GP) was calculated as GP (%) = (number of normal seedlings/number of seeds for testing) × 100. The germination index (GI) was calculated as GI = Σ (Gt/Dt), “Dt”—days to germination, “Gt”—number of germinated seeds per day corresponding to “Dt”; “Σ” is the sum. The vitality index (VI) was calculated as VI = GI × S; “S” is the dry weight (g) of buds in a certain period. The percentage decrease in GP (%) = ((GP of standard germination test method − GP of artificial accelerated aging method)/GP of standard germination test method) × 100. The percentage decrease in GI (%) = ((GI of standard germination test method − GI of artificial accelerated aging method)/GI of standard germination test method) × 100. The percentage decrease in VI (%) = ((VI of standard germination test method − VI of artificial accelerated aging method)/VI of standard germination test method) × 100.

### 4.4. RNA Extraction and Sequencing

Total RNA extraction was carried out using the Total RNA Rapid Extraction Kit (BioTeke, Beijing, China, product No. RP3302). Read count values were statistically calculated for each gene using HTseq (1.99.2), representing the basic expression of the gene, and standardized for each gene using fragments per kilobase of transcript per million mapped reads (FPKM) to make the genes comparable between the two cultivars and populations. DEGs were screened using DESeq (1.34.0) with a threshold of FDR < 0.05 and |log_2_ fold change| > 0.

### 4.5. Analysis of DEGs

To understand the functions of DEGs, we performed GO enrichment and KEGG enrichment analyses of DEGs using the “PlantNGSTools” R package. We considered GO and KEGG terms with a *p*-value < 0.05 to be considered significantly enriched. We used SIRING (https://string-db.org/, accessed on 1 February 2024) to analyze protein–protein interaction networks and Cytoscape v.3.10.1 to draw the regulatory network of target genes.

### 4.6. qRT-PCR Validation of DEGs

Six DEGs were randomly selected for this study and used to validate the DEG results. Total RNA was extracted as described in Section 4.4 and reverse transcribed for qPCR analysis using the HiScript II Q RT SuperMIX for qPCR (+gDNA wiper) kit (R223-01, Vazyme). The internal reference gene used was *Ubi* (*LOC_Os03g13170*, encodes ubiquitin fusion protein), and the amplification primers were designed using Primer Premier 6.0. The changes in gene expression levels were quantified using the 2^−ΔΔCT^ method with three biological replicates. Table S8 displays all primer information.

## 5. Conclusions

**Figure 7:**
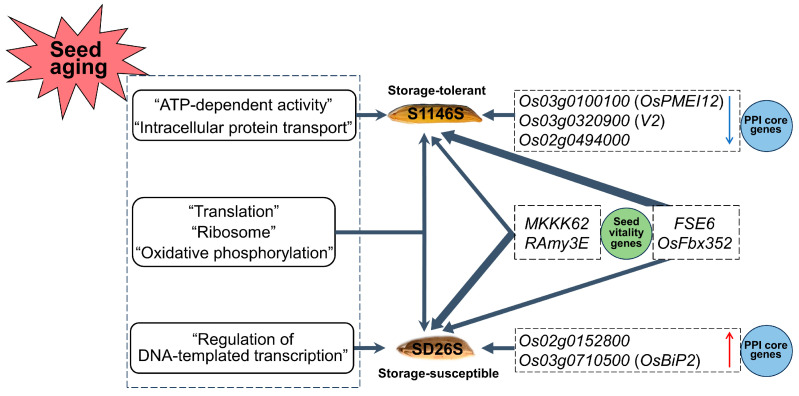
Schematic diagram of the storability regulation in S1146S and SD26S seeds during aging treatment. The ↑ in red indicates up-regulation, while the ↓ in blue indicates down-regulation. The thickness of the line indicates the level of expression, with thicker lines representing higher levels of expression.

In this study, we conducted an investigation into the transcriptome of the seeds of two rice TGMS lines, S1146S and SD26S, under 0 and 7 days of artificial accelerated aging treatment using RNA-seq. In total, 3447 DEGs were identified in S1146S and SD26S, and these DEGs were categorized into four groups (G1, G2, G3, and G4) based on their regulatory patterns. GO and KEGG enrichment analysis revealed that both lines shared “translation”, “ribosome”, and “oxidative phosphorylation”. However, DEGs related to “ATP-dependent activity” and “intracellular protein transport” were specifically enriched in S1146S, while “regulation of DNA-templated transcription” was particularly enriched in SD26S (Figure 7).

Several genes associated with seed storability were identified in G4, including *Os01g0971400, Os01g0937200*, and *Os07g0214300*, which are involved in “proteolysis”; *Os03g0276500*, related to “cellular response to unfolded protein”; and *Os05g0328632*, related to “vacuole”. Additionally, core genes of the PPI network, such as *Os03g0100100* (*OsPMEI12*), *Os03g0320900* (*V2*), and *Os02g0494000*, were found to be down-regulated in S1146S. These genes are associated with pectin esterase inhibitor activity, guanylate kinase activity, and binding, respectively. In SD26S, two unfolded protein response-related genes, *Os02g0152800* and *Os03g0710500* (*OsBiP2*), were up-regulated. Additionally, seed vitality-related genes, *MKKK62* (*Os01g0699600*), *OsFbx352* (*Os10g0127900*), *FSE6* (*Os05g0540000*), and *RAmy3E* (*Os08g0473600*), associated with seed storability were identified (Figure 7).

Overall, this study provides insights into the molecular response and related genes of TGMS lines under artificially accelerated aging conditions. These insights will provide new ideas for breeding hybrid rice varieties with strong seed storability.

## Data Availability

All raw sequencing reads generated in this study have been deposited in the public database of the National Genomics Data Center under data accession number PRJNA1078441. All the other data are available from the corresponding authors upon request.

## Supplementary Materials

The following supporting information can be downloaded at https://www.mdpi.com/article/10.3390/plants13070945/s1: Figure S1: qRT-PCR verification of expression profiles in RNA-seq. Figure S2: Expression profile of DEGs enriched in G4. Figure S3: GO terms with similar regulation patterns in DEGs of S1146S and SD26S. Table S1: Sequencing data and sample mapping rates. Table S2: The log_2_ fold change and FDR values of DEGs in G1, G2, G3, and G4. Table S3: GO enrichment for DEGs in G1. Table S4: GO enrichment for DEGs in G2. Table S5: GO enrichment for DEGs in G3. Table S6: GO enrichment for DEGs in G4. Table S7: KEGG pathway enrichment comparison among G1, G2, G3, and G4. Table S8: Primers used in qRT-PCR.
