# Supplementary material for: Transcriptomic Profiling of Two Rice Thermo-Sensitive Genic Male Sterile Lines with Contrasting Seed Storability after Artificial Accelerated Aging Treatment
Source: Plants (Basel). 2024 Mar 25;13(7):945. doi: 10.3390/plants13070945 (PMC11013862; doi:10.3390/plants13070945)
Supplement: Supplementary file 1 [file plants-13-00945-s001.zip › Supplementary figures.pdf]

Supplementary figures

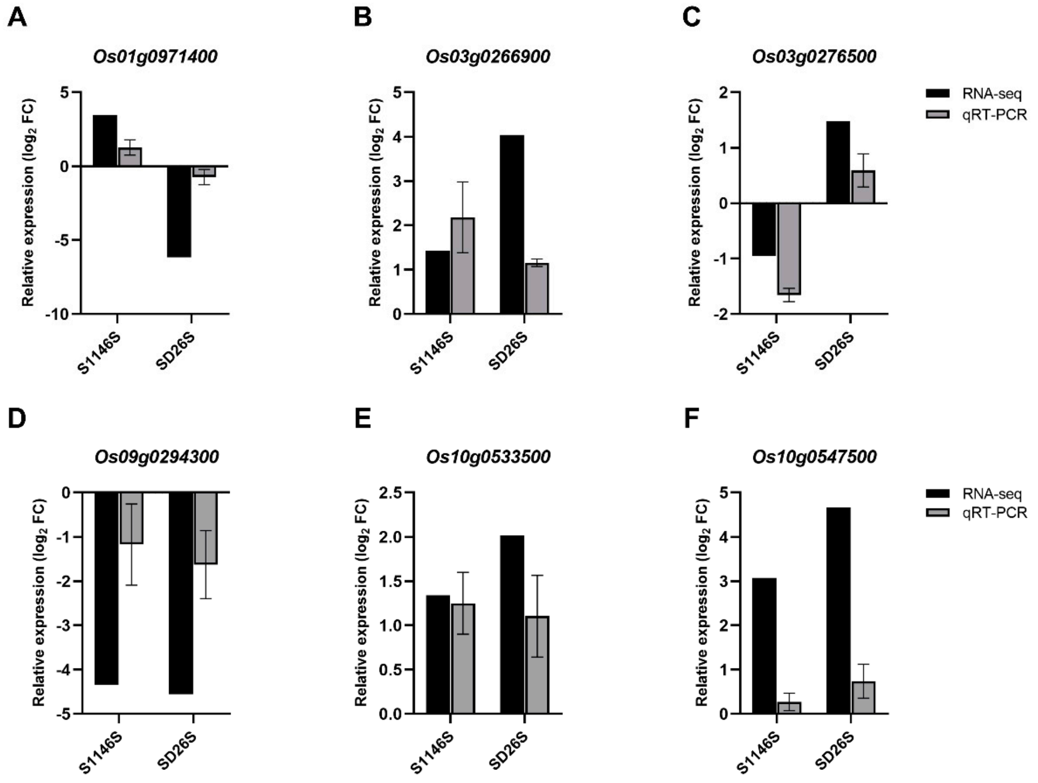

Figure S1. qRT-PCR verification of genes expression profiles in RNA-seq. Changes in gene expression levels (log_2 FC values) of (**A**) *Os01g0971400*, (**B**) *Os03g0266900*, (**C**) *Os03g0276500*, (**D**) *Os09g0294300*, (**E**) *Os10g0533500*, and (**F**) *Os10g0547500* in qRT-PCR and RNA-seq. Data are given as means ± SD, n=3.

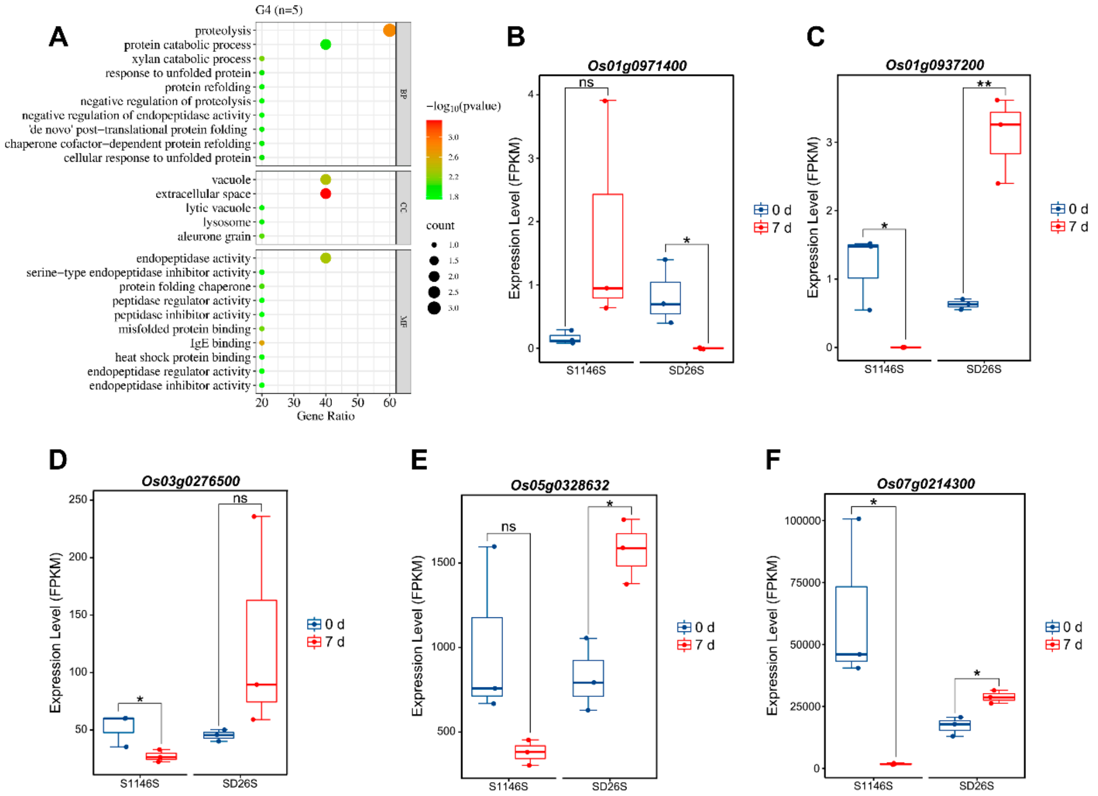

Figure S2. Expression profile of DEGs enriched in G4. (**A**) GO terms of DEGs (G4) that were conversely differentially expressed in both S1146S and SD26S. Comparative analysis of the expression levels (FPKM) of (**B**) *Os01g0971400*, (**C**) *Os01g0937200*, (**D**) *Os03g0276500*, (**E**) *Os05g0328632*, (**F**) *Os07g0214300*, between S1146S and SD26S. The data are presented as means ± SEM (standard error of the mean) from three independent replicates. Statistical significance is denoted as follows: ns (not significant) for $p \geq 0.05$, * for $p < 0.05$, ** for $p < 0.01$.

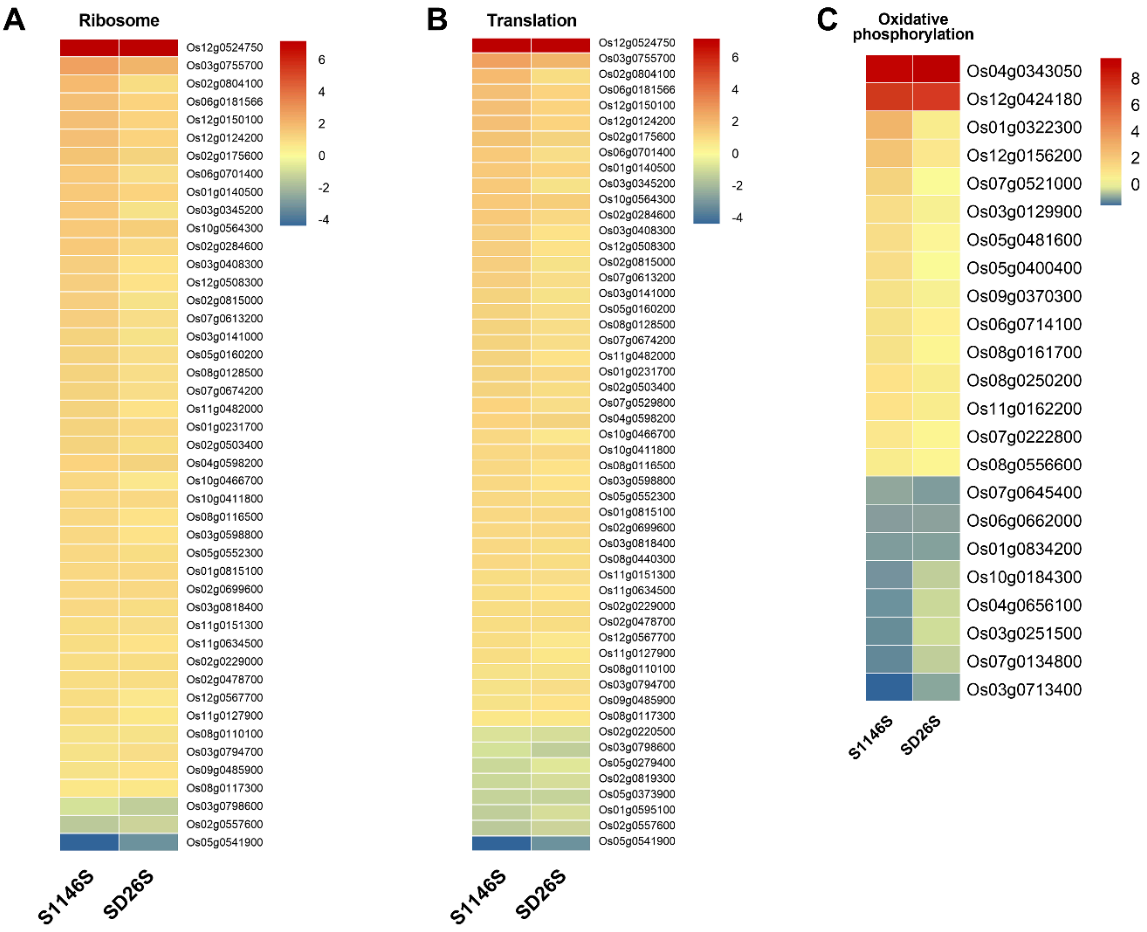

Figure S3. GO terms with similar regulation patterns in DEGs of S1146S and SD26S. Heatmap depicting the expression levels of the DEGs enriched in the (**A**) "Ribosome" term, (**B**) "Translation" term, and (**C**) "Oxidative phosphorylation" term. The heatmap is constructed based on $\log_2$ fold change values for all samples, with the blue to red color spectrum representing low to high levels of gene expression.
